# Development and Validation of a Tunable Diode Laser Absorption Spectroscopy System for Hot Gas Flow and Small-Scale Flame Measurement

**DOI:** 10.3390/s22176707

**Published:** 2022-09-05

**Authors:** Ran Tu, Junqing Gu, Yi Zeng, Xuejin Zhou, Kai Yang, Jiaojiao Jing, Zhihong Miao, Jianhong Yang

**Affiliations:** 1 College of Mechanical Engineering and Automation, Huaqiao University, Xiamen 361021, China; 2 Department of Modern Mechanics, University of Science and Technology of China, Hefei 230026, China

**Keywords:** tunable diode laser absorption spectroscopy (TDLAS), gas flow field, H_2_O-based TDLAS, doppler-shift, small-scale flame

## Abstract

TDLAS (tunable diode laser absorption spectroscopy) is an important gas analysis method that can be employed to obtain characteristic parameters non-invasively by the infrared absorption spectra of tracer molecules such as CH_4_, H_2_O and O_2_. In this study, a portable H_2_O-based TDLAS system with a dual optical path was developed with the aim of assessing the combustion characteristics of flammable gases. Firstly, a calculation method of gas characteristics including temperature and velocity combining absorption spectra and a HITRAN database was provided. Secondly, to calibrate and validate this TDLAS system precisely, a pressure vessel and a shock tube were introduced innovatively to generate static or steady flow fields with preset constant temperatures, pressures, or velocities. Static tests within environment pressures up to 2 MPa and steady flow field tests with temperatures up to 1600 K and flow velocities up to 950 m/s were performed for verification. It was proved that this system can provide an accurate values for high temperature and velocity gas flows. Finally, an experimental investigation of CH_4_/air flames was conducted to test the effectiveness of the system when applied to small diffusion flames. This TDLAS system gave satisfactory flame temperature and velocity data owing to the dual optical path design and high frequency scanning, which compensated for scale effects and pulsation of the flame. This work demonstrates a valuable new approach to thermal hazard analysis in specific environments.

## 1. Introduction

The measurement of hot gas characteristic parameters in complex environments—e.g., those involving high flow rates, vibration, or combustion—has posed a long-standing challenge to the study of aero-engines and industrial furnaces [1]. In recent decades, there has been positive exploration concerning the non-contact assessment of combustion fields using spectral imaging technologies, including emission, absorption, Raman spectrum, and LIF/PLIF (laser induced fluorescence/planar laser induced fluorescence). Of all these methods, TDLAS (tunable diode laser absorption spectroscopy) has emerged an effective yet inexpensive technique providing a rapid response [2]. 

TDLAS technology has undergone significant development attributable to the progress in semiconductor lasers. As a consequence, this technique is now widely used for the non-contact measurement of temperature, velocity, or species concentrations for environmental engineering, petroleum engineering, and hazardous chemicals detection. Additionally, when incorporating DFBs (distributed feedback lasers) and VCSELs (vertical cavity surface emitting lasers), a TDLAS system becomes more portable and easier to establish [3,4].

Early research regarding the application of TDLAS to the analysis of combustion was reported by Hanson and Allen et al. [1,5,6,7]. Characteristic parameters of hot gases in combustion flow fields involving high temperatures and pressures were assessed by TDLAS based on the absorption spectra of O_2_ and H_2_O. This method was then extended to the monitoring of waste gases such as NO_2_ and NH_3_ for the process control of industrial combustion systems. With the development of tunable diode laser sources, the useable wavelength range associated with this method was broadened to approximately 700–3300 nm, allowing the analysis of H_2_O, CO, CO_2_, CH_4_, and other gases [8,9]. The data obtained from TDLAS have provided new perspectives concerning the monitoring of combustion processes [10]. 

An effective component for flame measurement by TDLAS now well recognized is H_2_O [11,12,13]. The most important reason is that H_2_O is commonly one of the primary combustion products and can reflect the progress of combustion based on the local thermodynamic equilibrium hypothesis. Although the measurement by gas molecular absorption spectra with TDLAS seems to be a proven technology, there are still significant deficiencies related to this technique. Specifically: 

(1) Generally, a typical TDLAS analysis provides only the average value of a selected parameter along the optical path.

(2) Since it is challenging to generate a constant flow field with a given temperature, pressure, and velocity, the calibration of a TDLAS system is relatively difficult. 

(3) H_2_O-based TDLAS measurement can also be disturbed by the presence of ambient water vapor, leading to underestimations of temperature and overestimations of component concentrations.

(4) The Doppler effect can interfere with TDLAS velocity measurements in high-speed flow fields.

The aim of the study is to develop a portable TDLAS system for combustion analysis and to provide an associated means of calibrating this system. In this paper, a TDLAS system was designed and applied to the assessments of gas flow and CH_4_/air co-flow flame including temperature and velocity measurements. A static flow field vessel and a shock tube were employed to generate a steady flow field along with specific constant temperature or velocity for precise calibration. The measurement results obtained from this system were verified. Finally, experimental investigation of the CH_4_/air flame was conducted to test the effectiveness of the new TDLAS system in combustion diagnosis.

## 2. Experimental Methodology

### 2.1. Absorption Fundamentals

The principle of TDLAS measurement is on the basis of the Beer–Lambert law, depicting the relationship of incident light, transmitted light, molecule temperature, and concentration [14] as

(1) $$I(\nu )=I_{0}(\nu )e^{-\sum_{i}\alpha_{i}(\nu )L},\;\mathrm{and}\;\alpha_{i}(\nu )=p_{i}\varphi_{i}(\nu -\nu_{0})S_{i}(T)$$

where I and $I_{0}$ are the transmitted light intensity and incident light intensity, respectively, which are corresponding to the output frequency of laser, ν. $\alpha_{i}(\nu )$ is the absorptivity of absorbing species *i*# and L is the path length. Moreover for $\alpha_{i}(\nu )$, $p_{i}$, and $\varphi_{i}(\nu -\nu_{0})$ are the partial pressure and absorption line shape function of species *i*#, such as Gaussian, Lorentzian, or Voigt line profile ($\nu_{0}$ is the center frequency of the absorption spectrum) [15]. In addition, $S_{i}(T)$, as a function of temperature, is the molecular absorption line strength of species *i*#. Consequently, integrated absorbance of independent transition line can be obtained as

(2) $$A_{i}=\int_{-\infty }^{+\infty }-\ln(I/I_{0})dv=\int S_{i}(T)\varphi_{i}(\nu -\nu_{0i})d\nu$$

Based on the theory of molecular spectroscopy [16], the absorption line strength at temperature *T* can be expressed as

(3) $$S(T)=S(T_{0})\frac{Q(T_{0})}{Q(T)}\cdot \exp[-\frac{hcE}{k}(\frac{1}{T}-\frac{1}{T_{0}})]\cdot \frac{1-\exp(-hcE/kT)}{1-\exp(-hcE/kT_{0})}$$

where $T_{0}$ is a reference temperature. $\varepsilon_{0}$, h, c and k are four constant numbers, including the dielectric constant, Planck’s constant, speed of light and Boltzmann’s constant, respectively. E is the energy of the lower transition state, and Q(T) is the molecular internal partition function at temperature *T*, which could be obtained from the HITRAN database [17,18]. These relationships enable the non-invasive experimental analysis of hot gases. 

### 2.2. Measuring Principle of Gas Temperature and Velocity

**Figure 1:**
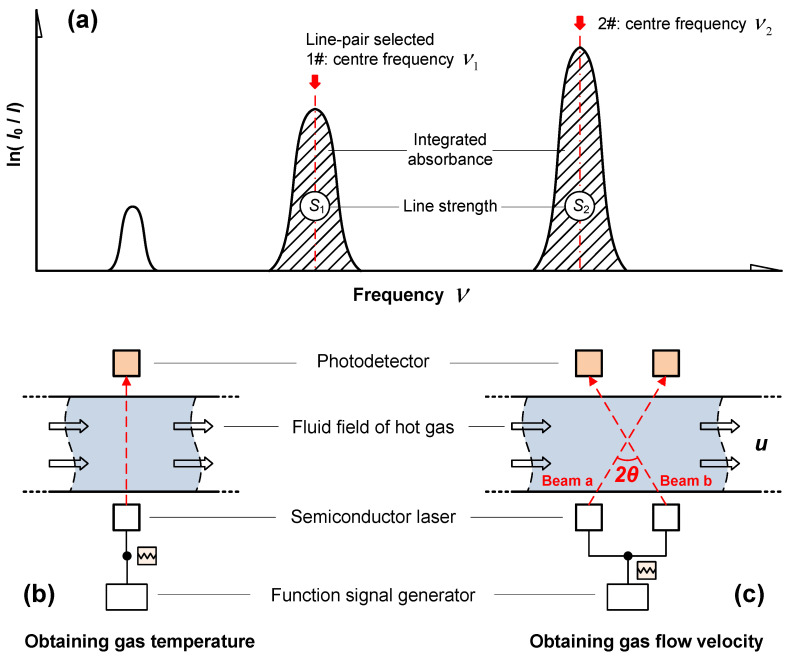
(**a**) Integrated absorbances of two selected transitions and diagrams summarizing the processes used to measure (**b**) gas temperature and (**c**) flow velocity.

The absorption line shape, bandwidth and intensity of absorption spectral features can be obtained using a TDLAS system together with wavelength-scanning. Furthermore, the gas temperature can be calculated from the ratio, *R* (the integrated absorbances of two transition [7]), as

(4) $$R=\frac{\int S_{1}(T)\varphi_{1}(\nu -\nu_{01})d\nu }{\int S_{2}(T)\varphi_{2}(\nu -\nu_{02})d\nu }=\frac{S_{1}(T_{0})}{S_{2}(T_{0})}\exp[-\frac{hc(E_{1}-E_{2})}{k}(\frac{1}{T}-\frac{1}{T_{0}})]$$

where subscripts 1 and 2 denote the two absorption transitions selected as shown in Figure 1a. $\Delta E=E_{1}-E_{2}$ is the energy separation of the absorbing states. Based on the use of a near-IR laser (Figure 1b), the gas temperature can then be obtained from the relationship [19]

(5) $$T=\frac{-\frac{hc}{k}\Delta E}{\ln R+\ln\frac{S_{2}(T_{0})}{S_{1}(T_{0})}-\frac{hc}{kT_{0}}\Delta E}$$

On the other hand, the gas flow velocity can be calculated according to Doppler-shift as shown in Figure 1c [20]. Assuming that a monochromatic laser beam with a frequency $\nu_{0}$ (the same as the center frequency of the absorption spectrum) is used here, the Doppler-shifted center frequencies for laser beams a and b would change into

(6) $$\nu_{1}=\frac{\nu_{0}c}{c+u\sin\theta }$$

(7) $$\nu_{2}=\frac{\nu_{0}c}{c-u\sin\theta }$$

Considering that c>>u, the flow velocity relationship can be simplified to

(8) $$\Delta \nu =\nu_{2}-\nu_{1}=\frac{2u\nu_{0}c\cdot \sin\theta }{c^{2}-u^{2}\sin^{2}\theta }\sim \frac{2u\nu_{0}\cdot \sin\theta }{c}$$

(9) $$\mathrm{or}\;u=\frac{c\cdot \Delta \nu }{2\nu_{0}\cdot \sin\theta }$$

### 2.3. The Experimental TDLAS System

**Figure 2:**
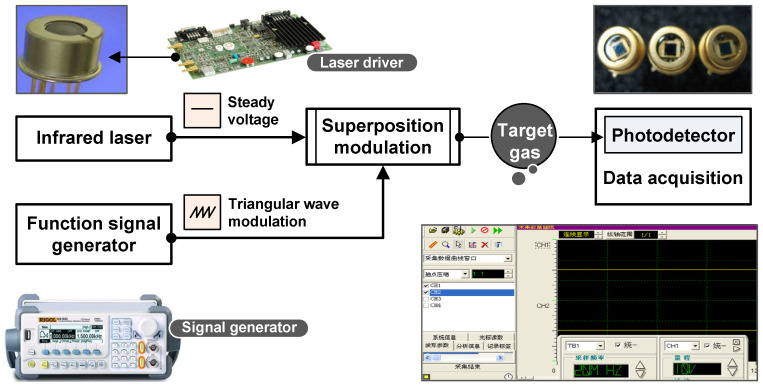
Schematic of the TDLAS system designed in the present work.

The TDLAS system designed in this work mainly comprised an infrared laser, modulation unit, optical path, photodetectors, and high-speed data acquisition and processing module, as shown in Figure 2. An OEM VCSEL driver (VITC002 from Thorlabs, Newton, NJ, USA) with a temperature controller was applied for laser modulation. The functional parameters of the laser (VCSEL from Vertilas, München, Germany) and function signal generator (DG-1022 from Rigol, Beijing, China) are listed in Table 1.

Because of the advantages provided by analyzing water vapor, H2O-based TDLAS measurement assessments were used in this study but with more accurate experimental validation and a specially designed optical path intended for the monitoring of combustion processes. Water will produce intense absorbance bands in the near-IR region 1400, 1800, and 2700 [16,21]; and to avoid any interference by other species (such as C-H radical), 1392 nm was selected as the center wavelength for water vapor detection. Photodetectors (PN-2000 from Lightsensing Technologies, Beijing, China) with a response range of 900–1650 nm were used to determine the transmitted light intensity. Data were obtained using a data acquisition card (PCI-20612 from TDEC, Sichuan, China) with four channels, operating at 32 bits and a maximum rate of 50 MSa/s. 

### 2.4. Experimental Design for Validation

**Figure 3:**
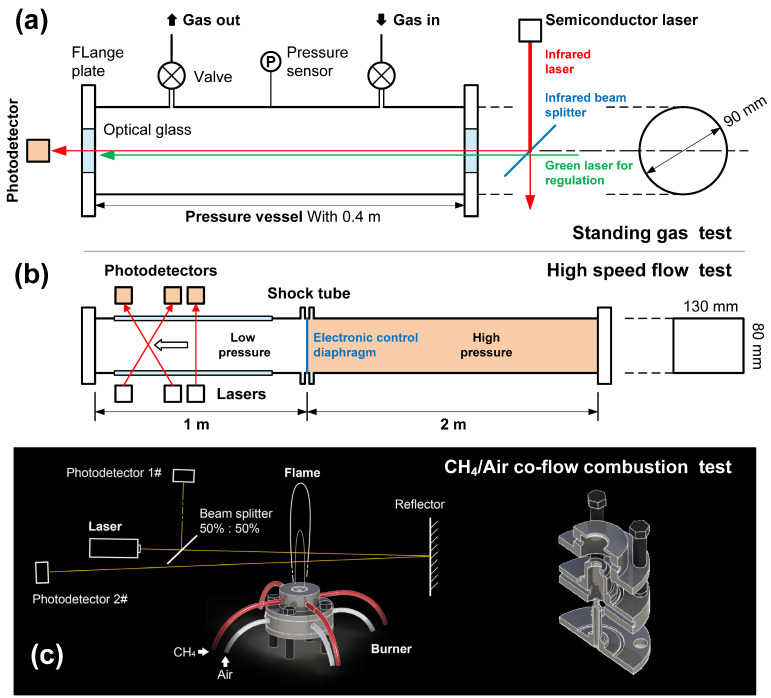
Experimental facilities for TDLAS tests, including (**a**) a pressure vessel, (**b**) a shock tube, and (**c**) a co-flow flame burner.

The functioning of the TDLAS system was calibrated or examined in three ways, as shown in Figure 3, using a pressure vessel, a shock tube, and a co-flow combustion platform. 

(1) Firstly, normal pressure and temperature were applied to the pressure vessel (Figure 3a) with standing air to calibrate the basic performance of the system. This vessel was made of stainless steel with optical glasses on both sides. The optical path in this device had a maximum length of 0.4 m and a 532 nm green laser was employed to adjust the path.

(2) Secondly, the shock tube was intended to provide determinable high-temperature and high-speed water vapor flow to permit the precision and response rate of the measurement system to be ascertained. As shown in Figure 3b, the shock tube was comprised a high-pressure section, a low-pressure section, a gas circuit, and an electronically controlled diaphragm. Prior to each test, the low-pressure section was charged with air to a preset pressure. Following this, the high-pressure section was also slowly filled with air until the diaphragm instantaneously ruptured to create a shock wave, thus producing a high-temperature/pressure, high-speed flow field.

(3) Finally, the calibrated TDLAS system was used for the CH_4_/Air flame temperature and hot gas velocity measurements, as shown in Figure 3c. A co-flow CH_4_/air burner was made to generate a stable diffusion flame with preset initial conditions [22,23]. High precision mass flowmeters (KM7100 from Alicat, Tucson, AZ, USA) were used to dispense the combustible gases. To avoid the disturbance by H_2_O absorption in the non-flame zone (i.e., a background signal resulting from atmospheric H_2_O), a beam splitter (50%:50%) was used to subtract the background interference. As noted, the flame width (absorption length about 2–3 cm) was relatively short, thus a reflector was added to obtain a stronger absorption signal.

The experimental conditions are summarized in Table 2. All the tests were repeated 20 times to ensure reproducible results.

## 3. Results and Analysis

### 3.1. Room Temperature Measurement by TDLAS

**Figure 4:**
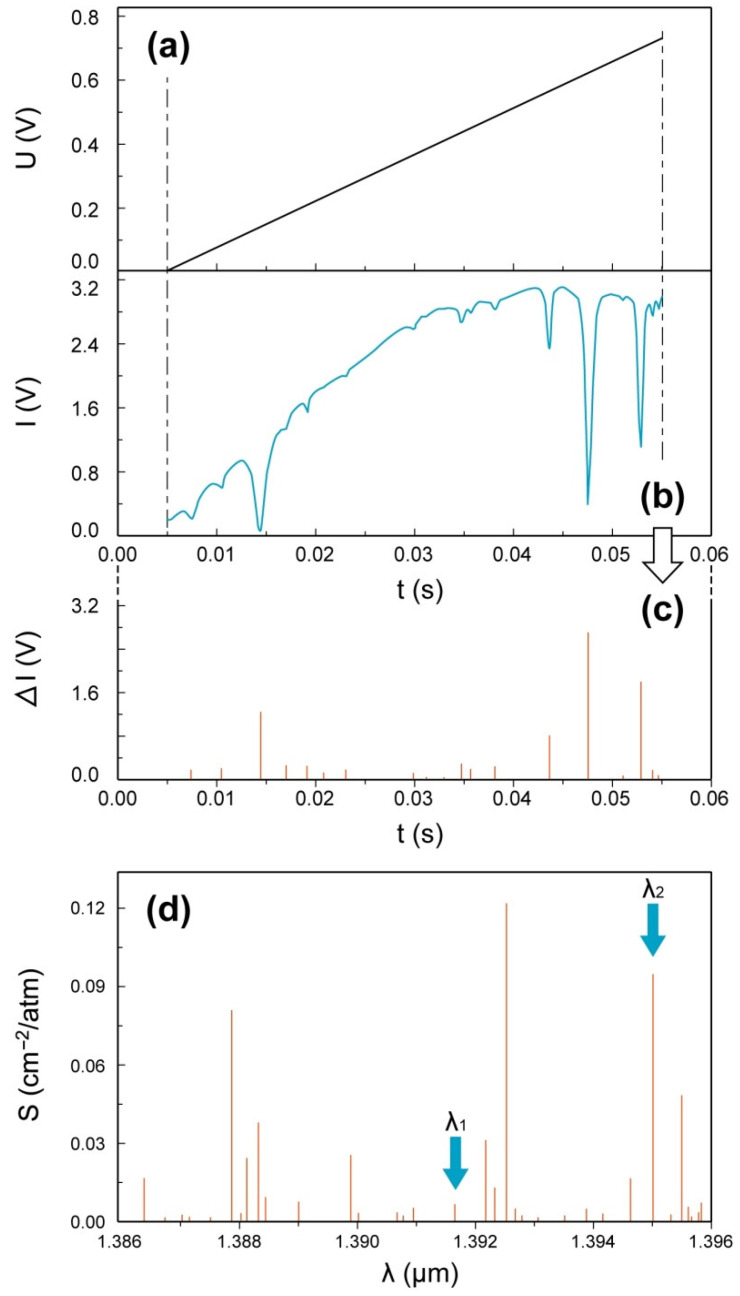
Variation in (**a**) laser driving voltage and (**b**) transmitted light intensity in a half circle with (**c**) peak positions and strengths, and (**d**) the line strength distribution calculated from the HITRAN [17] database.

Absorption spectra of the contents of the pressure vessel (see Figure 3a) could be obtained on the basis of comparisons between the laser output and absorption line strength using the HITRAN [17] data, as shown in Figure 4 with an example at initial pressure of 1 atm. Figure 4a shows the voltage *U* variation of function signal generator output used for driving laser during a half cycle, and Figure 4b presents the transmitted light intensity after absorption. Furthermore, clear positions and strengths of absorption peaks could be found in Figure 4c. The line strength of water vapor vs. wavelength is plotted in Figure 4d with independent absorption line.

To calculate the vapor temperature, the time-domain of transmitted light intensity (*I* vs. *t*) should be transformed to frequency-domain (that is, *I* vs. ν or *I* vs. λ) at first. Based on the approximately linear relationship between λ and *U*, two reference wavelength-time points were selected: ($\lambda_{1}$, $t_{1}$) and ($\lambda_{2}$, $t_{2}$). Then we obtained

(10) $$\lambda =\frac{t-t_{2}}{t_{1}-t_{2}}(\lambda_{1}-\lambda_{2})+\lambda_{2}$$

This simplified linear fitting was considered a reasonable approximation over short time spans. Note that λ vs. *t* would not be a continuous function, due to the discreteness of λ. Furthermore, the wavelengths that were selected for these calculations ($\lambda_{1}$ and $\lambda_{2}$) should be a certain distance apart to reduce the error caused by uncertainties in determining the positions of the absorption peaks. Hence, $\lambda_{1}$ = 1391.67275 nm and $\lambda_{2}$ = 1395.00424 nm were selected in the present work for the purpose of wavelength calibration.

**Figure 5:**
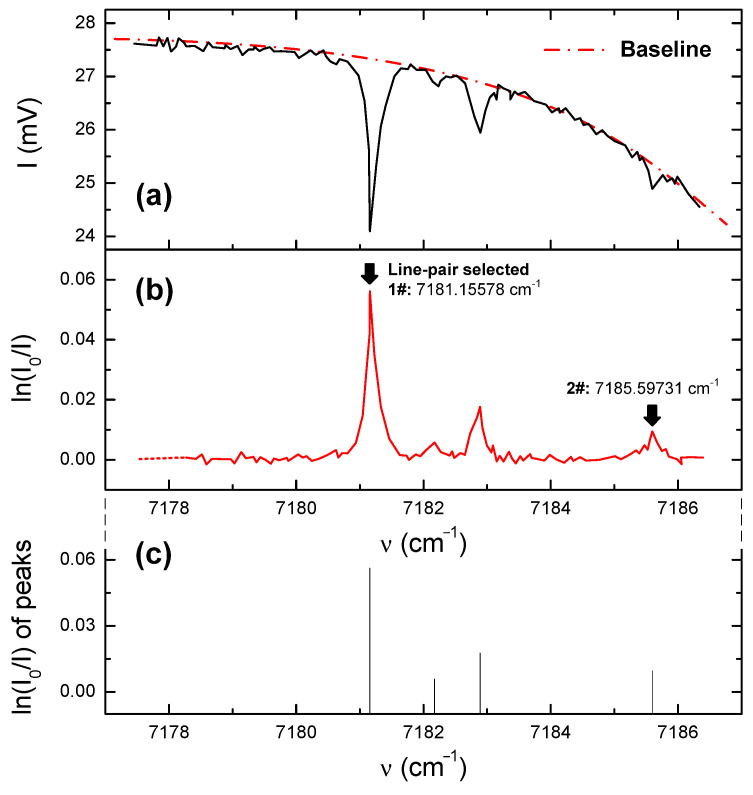
(**a**) Absorption frequency-domain diagrams of the transmitted light intensity and (**b**) the absorption ratio with (**c**) main peak positions and strengths.

Consequently, as ν~1/λ, the correlation between *I* and ν could be obtained by combining Equation (10) with the data in Figure 4, as shown in Figure 5a. The baseline in Figure 5a was fitted by using the polynomial $I_{0}=a_{0}+a_{1}\nu +a_{2}\nu^{2}+a_{3}\nu^{3}$ and employing data within the non-absorption region. The curve of $\ln(I/I_{0})$ vs. ν, as the key relation for temperature calculation deduced in Equations (4) and (5), could be further illustrated in Figure 5b,c.

It is necessary to take into account that the line-pair selection had to meet certain conditions, meaning that there was no interference by other spectral lines and these lines were positioned near the central wavelength of the laser. Furthermore, the lines had to be separated by a suitable distance to avoid overlap. Therefore, we chose ν = 7181.15578 cm^−1^ and ν = 7185.59731 cm^−1^ under overall consideration. Other important parameters related to the HITRAN database [17] are provided in Table 3. Combining Equations (4) and (5) provide T = 302 ± 1.4 K, and this value—compared with the average experimental value of 301.14 ± 0.8 K by the thermocouples—provides a measurement error of less than 0.3%.

The pressure effects on measurement accuracy were investigated subsequently. The results showed that, with the enlarged initial test pressure, pressure broadening occurred generally and became dominant due to the increasing frequency of molecular collisions. In addition, spectral interference resulting from line overlap became evident at pressures exceeding 0.8 MPa. It should be noted that the measurement errors related to temperature and concentration could be larger than 10% at pressures above 1 MPa.

### 3.2. Velocity of High Speed Air Flow in Shock Tube

**Figure 6:**
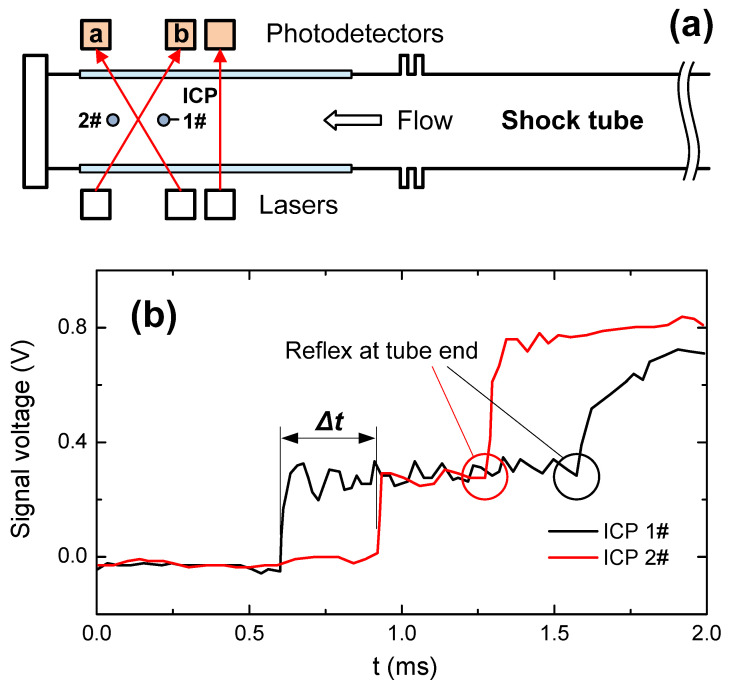
(**a**) ICP sensor locations and (**b**) responses for a pressure wave in the shock tube.

As shown in Figure 3b, a uniform and controllable air flow field could be generated in the shock tube. The velocity values measured by the TDLAS system were compared with those by both ICP (integrated circuits piezoelectric) shock wave pressure sensors (102B15 from Dibeiqi Electronic Technology, China) and theoretical calculations. Two ICP sensors with a distance of 120 mm were mounted along the tube as shown in Figure 6a, and typical results were plotted in Figure 6b. The shock wave velocity was determined from these data as

(11) $$u_{shock}=\frac{120\;\mathrm{mm}}{\Delta t_{ICP}}$$

Based on assuming isentropic flow, the flow velocity could be simplified as

(12) $$u=M\sqrt{\gamma R_{gas}T}$$

where *M* is the Mach number of the shock wave determined by $u_{shock}$ in Equation (11) and sound velocity, γ is the adiabatic exponent, and *R_gas_* is the universal gas constant.

**Figure 7:**
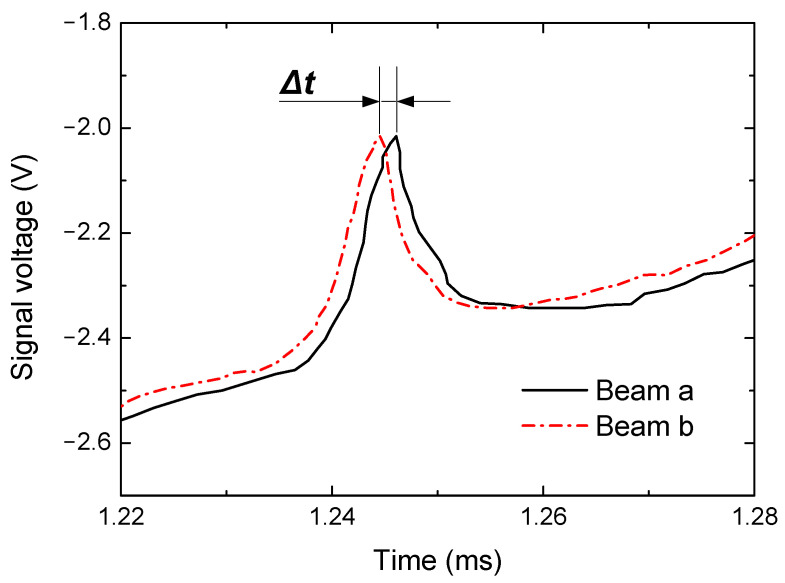
Time difference in the TDLAS signal due to the frequency shift induced by the Doppler effect.

The signals obtained from laser beams a and b during the TDLAS analyses are shown in Figure 7. To avoid miscalibaration, the synchronization of the initial absorption peaks of the two lasers under static air condition were performed. According to the correlation of *t* with λ (or Δt vs. Δλ) in Equation (10), the frequency shift resulting from the Doppler effect could be

(13) $$\left|\Delta \nu \right|=\left|\Delta \frac{1}{\lambda }\right|\sim \frac{\left|\Delta \lambda \right|}{\lambda_{0}^{2}}$$

and combining this relationship with Equation (9) allowed the flow velocity to be determined from the TDLAS results.

In theory, the flow velocity and temperature could also be predicted by shock wave propagation equations numerically [24]. It is helpful to summarize the characteristics of the three measurement methods:

(1) The ICP sensors can capture the shock wave movement and time interval, which are the key parameters to calculate flow velocity with shock wave theory.
(2) As noted above, the velocity by TDLAS measurement is actually that of H_2_O molecule.
(3) Numerical prediction is based on both the Mach number (obtained according to the pressure ratio between the high-pressure and low-pressure sections) and the shock wave propagation equations.

**Figure 8:**
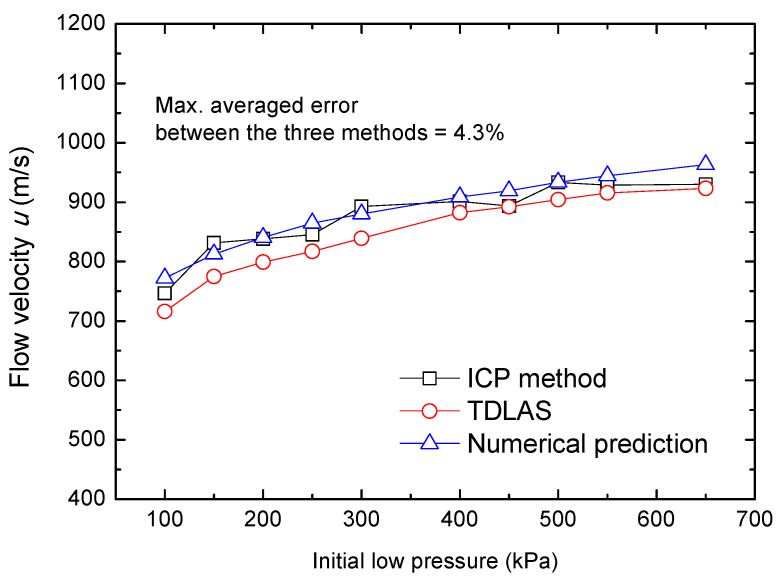
Gas flow velocities in the low-pressure section of the shock tube as determined using three methods.

To validate these methods, tests were conducted applying an initial pressure in the range of 100–650 kPa for the low-pressure section, and a comparison of the results is provided in Figure 8.

It is found that the results obtained from the ICP sensors and the numerical predictions were similar, presumably because both methods are on the basis of Mach number calculations and shock wave theory. However, the manner in which the Mach number is obtained is very different between the two. In the case of the ICP sensors method, the Mach number was deduced by shock wave velocity and sound velocity of the wave front, whereas, for numerical prediction, the Mach number was calculated from the aforementioned pressure ratio and iterative solution of gas dynamics relationships. Moreover, we should note that some assumptions had been made in shock wave theoretical analysis that may have led to a slight overestimation of the flow velocity. Specifically,

(1) Air viscosity effects and the propagation of waves in more than one dimension were ignored.

(2) The attenuation of the shock wave along shock tube was not considered.

(3) Considering the velocity range of gas flow, the re-absorption effect was also ignored [25].

**Figure 9:**
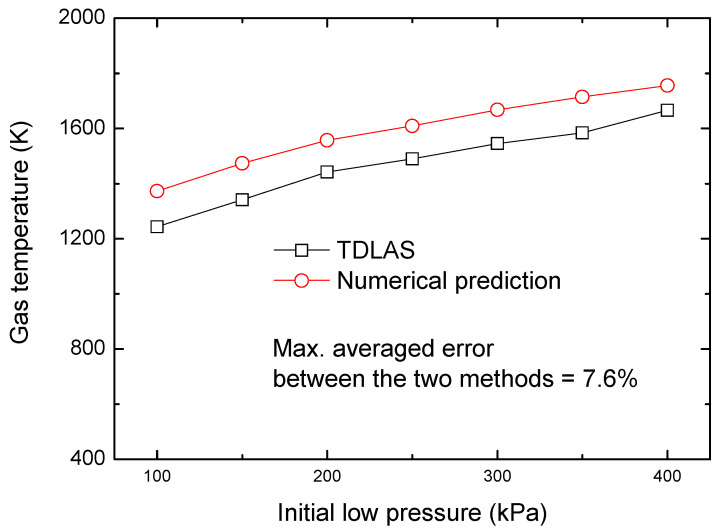
Comparison of gas temperature after reflected shock wave by two measurement methods.

In general, for high-speed gas flow velocity, the TDLAS measurements show good agreement the results obtained using the other two methods, with a maximum averaged error of 4.3%. The temperature of the reflected shock wave was also monitored, and a comparison between the TDLAS results and numerical predictions is shown in Figure 9. It is proved that the TDLAS system we developed in the present work was able to accurately capture the variation of temperature and velocity for high-speed gas flow.

### 3.3. Local Temperature and Velocity of CH_4_/Air Flame 

**Figure 10:**
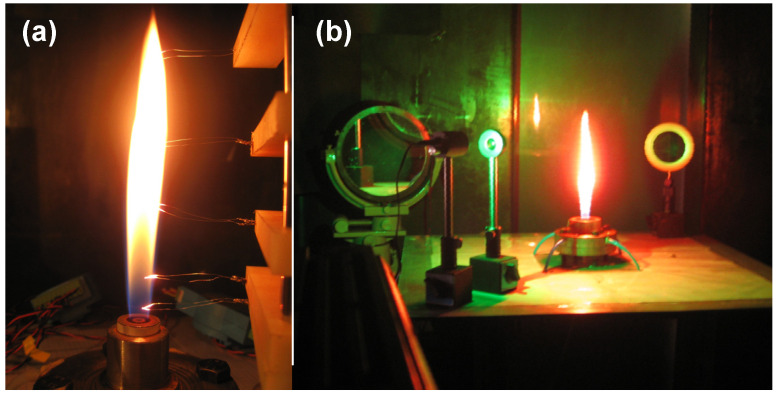

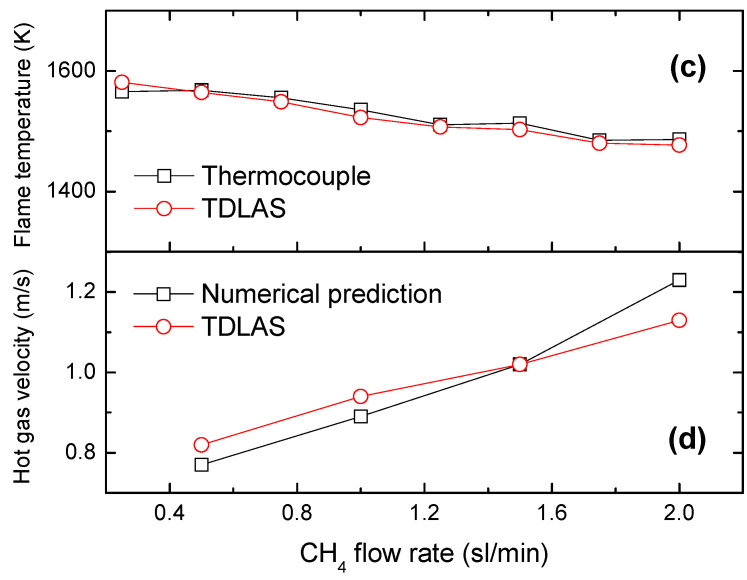
CH_4_/air flame data acquired using (**a**) thin Pt-Rh thermocouples, (**b**) the TDLAS system, and validations of the (**c**) flame temperature and (**d**) velocity results.

As one of the most important combustion products, the H_2_O is an ideal tracer for flame measurement by the TDLAS system. Here, variations in S(T) were evaluated using a thin Pt–Rh thermocouple wire with a diameter of 0.1 mm to estimate the approximate flame temperature range (in the vicinity of 1500 K). These data were subsequently used to validate the results by TDLAS as shown in Figure 10a,b.

The line-pair of ν = 7181.15578 cm^−1^ and ν = 7185.59731 cm^−1^ was again used in this assessment. During the trials, the fuel mass (or volume) flow rate of CH_4_/air diffusion flame was controlled by mass flowmeters to obtain an air flow rate of 2 standard liters per minute (slpm) and CH_4_ flow rates in the range of 0.25–2 slpm under an ambient pressure of 1 atm.

Figure 10c compares the temperature results between the thermocouples and the TDLAS system. The tendencies and actual values of the two curves are very similar, meanwhile, velocities by numerical simulations and the TDLAS process are also compared in Figure 10d with good agreement. It is interesting that although there were pulsations of the flame during testing, it can be ignored when using a driver scanning frequency above 1 kHz, meaning that approximately steady state flame information could be acquired at high scanning and sampling frequencies.

## 4. Conclusions

With the goal of performing combustion diagnostics and assessing the thermal characteristics of flammable gases, a portable H2O-based TDLAS system was designed. The measuring principle was revealed theoretically, and experimental calibration and validation were performed using a pressure vessel, shock tube, and CH_4_/air diffusion flame, respectively. The main conclusions from this work are:

(1) A method of calculating gas characteristics including temperature, velocity, and species concentration was provided using H_2_O absorption spectra and the HITRAN database in conjunction with TDLAS system. In addition, the line-pair selection criteria was verified. This method was demonstrated to be a viable approach to hot gas analyses. 

(2) The dual optical path TDLAS system established can eliminate the background interference effectively when applied to a relatively small test target. The combined selection of an appropriate center wavelength, wavelength calibration between the time and frequency domains, and use of a specific scanning or sampling frequency allowed this system to be used for the assessment of small flames.

(3) The temperature calibration and pressure broadening effect of absorption spectra of this TDLAS system were studied using a pressure vessel. It is found that this system can provide an accurate measurement within environment pressure of 0.8 MPa. 

(4) A shock tube was built to provide controllable and steady gas flows with high temperature or high speed, which turned out to be an ideal experimental setup for the parameter calibration of TDLAS at extreme conditions. The TDLAS system was confirmed to accurately monitor variations in high temperature and velocity gas flows.

(5) A small scale CH_4_/air diffusion flame burner was developed to validate the ability of the TDLAS system to monitor combustion characteristics. A comparison of the TDLAS results with thermocouple measurements and numerical simulations indicated that the TDLAS method provided satisfactory flame temperature and velocity values.

Future work will involve the application of TDLAS to the assessment of hydrocarbon combustion products such as CO_2_ and C-H radicals to explore more precise measurements.

## Figures and Tables

**Table 1:** Main parameters of laser and function signal generator selected.

Infrared Laser (VCSEL from Vertilas)	
Center wavelength (nm)	1392 *
Operating current range (mA)	0.5–8.5
Operating temperature range (°C)	15–35
Wavelength range at 20 °C (nm)	1389.16–1392.96
Wavelength range at 30 °C (nm)	1390.20–1393.70
Function signal generator (DG-1022 from Rigol)	
Maximum output frequency (MHz)	20
Frequency of sampling (MSa/s)	100
Frequency resolution (Hz)	1μ

* Center wavelength is chosen according to the absorption spectrum of H_2_O, which will be discussed later.

**Table 2:** Experimental conditions for tests by three facilities.

No.	Test Facility	Object	Initial Conditions
1	Pressure vessel	Air temperature	Room temperature Pressure: 90 kPa–2 MPa
2	Shock tube	Flow velocity	Velocity: 500–950 m/s
3	Combustion platform	Flame temperature and velocity	Room temperature at 1 atm CH_4_ flow rate: 0.2–2.0 sl/min

**Table 3:** Parameters queried and calculated from HITRAN.

Frequency (cm^−1^)	Absorption Line Strength under Room Temperature (cm^−2^/atm)	Energy of the Lower Transition State (cm^−1^)
7181.15578	0.12280	136.7617
7185.59731	0.00648	1045.0579

## Data Availability

Data is contained within the article.

## Nomenclature

A	Integrated absorbance of independent transition line
c	Speed of light (m s^−1^)
E	Energy of the lower transition state (J)
I	Signal of light intensity (V)
L	Path length (m)
p	Partial pressure (Pa)
S	Molecular absorption line strength (cm^−2^ atm^−1^)
T	Temperature (K)
t	Time (s)
u	Velocity (m s^−1^)
α	Absorptivity of absorbing species
ν	Frequency of laser
φ	Absorption lineshape function of species
θ	Angle between two lasers
λ	Wavelength
